# Urinary BA Indices as Prognostic Biomarkers for Complications Associated with Liver Diseases

**DOI:** 10.1155/2022/5473752

**Published:** 2022-03-30

**Authors:** Wenkuan Li, Jawaher Abdullah Alamoudi, Nagsen Gautam, Devendra Kumar, Macro Olivera, Yeongjin Gwon, Sandeep Mukgerjee, Yazen Alnouti

**Affiliations:** ^1^Department of Pharmaceutical Sciences, College of Pharmacy, University of Nebraska Medical Center, Omaha, NE 68198, USA; ^2^Department of Internal Medicine, College of Medicine, University of Nebraska Medical Center, Omaha, NE 68198, USA; ^3^Department of Biostatistics, College of Public Health, University of Nebraska Medical Center, Omaha, NE 68198, USA; ^4^Department of Internal Medicine, College of Medicine, Creighton University Medical Center, Omaha, NE, USA

## Abstract

Hepatobiliary diseases and their complications cause the accumulation of toxic bile acids (BA) in the liver, blood, and other tissues, which may exacerbate the underlying condition and lead to unfavorable prognosis. To develop and validate prognostic biomarkers for the prediction of complications of cholestatic liver disease based on urinary BA indices, liquid chromatography-tandem mass spectrometry was used to analyze urine samples from 257 patients with cholestatic liver diseases during a 7-year follow-up period. The urinary BA profile and non-BA parameters were monitored, and logistic regression models were used to predict the prognosis of hepatobiliary disease-related complications. Urinary BA indices were applied to quantify the composition, metabolism, hydrophilicity, and toxicity of the BA profile. We have developed and validated the bile-acid liver disease complication (BALDC) model based on BA indices using logistic regression model, to predict the prognosis of cholestatic liver disease complications including ascites. The mixed BA and non-BA model was the most accurate and provided higher area under the receiver operating characteristic (ROC) and smaller akaike information criterion (AIC) values compared to both non-BA and MELD (models for end stage liver disease) models. Therefore, the mixed BA and non-BA model could be used to predict the development of ascites in patients diagnosed with liver disease at early stages of intervention. This will help physicians to make a better decision when treating hepatobiliary disease-related ascites.

## 1. Introduction

Cholestatic liver diseases is a diverse group of hepatobiliary diseases associated with limitations in bile flow due to a failure of bile flow or an impairment in bile production [1]. Relatively common cholestatic liver diseases include primary biliary cirrhosis (PBC) [2], primary sclerosing cholangitis (PSC) [2], and alcoholic liver diseases [3].

Common complications associated with cholestatic liver diseases include ascites [4], bacterial peritonitis [5], encephalopathy [6], GI bleeding [7], hepatobiliary carcinoma [8], hepatorenal syndrome [9], jaundice [10], peripheral edema [11], and portal hypertension [12]. In particular, ascites is one of the most common complications associated with cirrhosis [13]. The risk of developing ascites is around 60% if the cause of cirrhosis has not been treated [14]. Cirrhosis is an advanced-stage liver disease caused by fibrosis, which impedes the intrahepatic blood flow, increases portal blood pressure, and causes accumulation of fluids in the peritoneal cavity (ascites) [15]. The survival of cirrhosis patients decreases from 80% to 50% when these patients are diagnosed with ascites [16]. Cirrhosis patients with ascites experience several symptoms, such as nausea [17], abdominal distention [18], dyspnea [19], edema [11], and hepatorenal syndrome [20].

Aspartate transaminase (AST), alanine transaminase (ALT), alkaline phosphatase (ALP), glutamyl transferase (GGT), serum creatinine, protime, and INR (international normalized ratio) are commonly used biomarkers for the diagnosis and prognosis of liver diseases [21–24]. However, these biomarkers are not specific to bile duct or liver injuries, and may be related to nonhepatobiliary conditions [21]. Therefore, models with multivariate parameters/markers were developed to better predict the prognosis of liver diseases with higher accuracy than individual parameters [25, 26].

Models with multivariate parameters are used to predict survival of hepatobiliary disease-related complications such as the Child-Turcotte-Pugh (CTP) and the Mayo model for end-stage liver disease (MELD) scores. The CTP score was originally used to determine the risk of shunt surgery for severity of liver disease and its complications, such as GI bleeding and encephalopathy [27, 28]. The MELD score was originally used to estimate survival of liver patients undergoing the transjugular intrahepatic portosystemic shunt (TIPS) [29]. The MELD score is currently used to determine patients' eligibility for liver transplantation [30, 31]. In addition, the MELD score is used as a predictor of liver disease complications, such as GI bleeding and portal hypertension [27, 29]. Even though the CTP and MELD scores are widely used worldwide, they still have several limitations. Variables of ascites and encephalopathy are easily affected by extraneous factors in the CTP score [29], while the MELD score has a poor evaluation for patients with cholestatic liver disease-related complications, such as ascites and encephalopathy [25].

More recently, bile acids (BA) have been considered as potential biomarkers for prognosis of hepatobiliary diseases [1, 32, 33]. BA are synthesized in the liver and excreted into bile, which flows to the small intestine via the bile duct [34]. BA have many physiological functions, such as fat absorption and cholesterol elimination [35]. Compared to their physiological functions, BA also exhibit pathological effects at high BA concentrations. They are associated with necrotic effects on mitochondria, detergent effects on biological membranes, and cancer promoting effects [36, 37]. There are a plethora of human and animal studies illustrating the link between the accumulation of toxic BA in the liver, blood and extrahepatic tissues, and unfavorable liver disease prognosis [1, 32, 38, 39].

However, BA have not been widely used in the clinic as biomarkers for liver diseases due to several limitations. Both individual and total BA concentrations have high inter- and intravariability under normal conditions due to several factors including weight, gender, and alcohol consumption, food ingestion, diurnal variation, and medication intake. Therefore, the normal baseline ranges are difficult to establish [40–44].

To address these limitations, we have established the concept of “BA idices.” BA indices are ratios calculated from the absolute individual BA concentration and their metabolites [1, 32, 45, 46]. BA indices have markedly low inter- and intraindividual variability and are more resistant to the above-mentioned cofactors than absolute BA concentrations. For example, the absolute total and individual BA concentrations increased more than 2-fold in individuals one hour after eating, while BA indices changed less than 10% in the same individuals [32]. Furthermore, we have demonstrated that urinary BA indices outperformed the currently used blood liver enzymes as biomarkers for cholestatic liver diseases [1, 32, 47]. In addition, we have recently developed a BA-based survival model (the BA score (BAS) model) to predict the prognosis of cholestatic liver diseases [48]. BAS had a higher true-positive and true-negative prediction of 5- and 3-year death and liver transplant than other non-BA models including MELD.

Multivariate markers and models are used to predict the survival of cholestatic liver diseases [49, 50]. However, very few studies have addressed the prognosis of cholestatic liver disease-related complications. For example, the CTP score has widely been used in the prognosis of cirrhosis, but it does not provide clear guidance of prognosis for cirrhotic patients with complications [51]. Similarly, the MELD score has extensively been used to prioritize cirrhotic patients awaiting liver transplantation [52], but it does not correlate with cirrhosis-related complications, including encephalopathy and bacterial peritonitis [53]. Therefore, there is a critical need for markers/models to particularly predict complications of liver diseases.

In this study, we have expanded the application of BA indices to predict complications, especially ascites, in patients with liver diseases. The study focuses on developing prognostic models based on BA indices to predict the development of ascites in liver patients.

## 2. Materials and Methods

### 2.1. Study Participants

The study population was described in details previously (cite our most recent paper [1, 32, 46, 48]. Briefly, patients with hepatobiliary conditions were diagnosed by University of Nebraska Medical Center's (UNMC) hepatology Clinic (Omaha, NE, USA). The institutional review board (IRB) approved this study at UNMC. Hepatobiliary conditions included chronic hepatitis C (64), chronic hepatitis B (15), Laennec's cirrhosis (105), primary biliary cholangitis (PBC) (12), primary sclerosing cholangitis (PSC) (15), alpha-1-antitrypsin deficiency (5), and cryptogenic cirrhosis (11). The following complications were diagnosed and monitored by the hepatologists: ascites (62), bacterial peritonitis (2), encephalopathy (36), GI bleeding (18), hepatobiliary carcinoma (15), hepatorenal syndrome (1), and portal hypertension (106). Two-hundred fifty-seven patients with cholestatic liver diseases between the ages of 19 and 65 years (121 female and 136 male) were recruited and treated at the UNMC from November of 2011 to December of 2018 into the study. Thirty milliliters' urine samples were collected from patients on their first and follow-up visits to the hepatology clinic. All urine samples were stored at -80°C before BA analysis using liquid chromatography-tandem mass spectrometry (LC-MS/MS) until analyzed. The study was approved by the institutional review board (IRB) at UNMC and written informed consents were provided for all participating subjects. The registry URL was (https://www.clinicaltrials.gov/ct2/show/NCT01200082?term=alnouti&draw=2&rank=1). The clinical trial number was NCT01200082.

### 2.2. Non-BA Parameters

The performance of potential biomarkers from the urinary BA profile has also been compared with the performance of existing markers of liver function including alanine transaminase (ALT), aspartate transaminase (AST), serum creatinine, albumin, bilirubin, international normalized ratio (INR), protime, AST/ALT ratio, and AST/platelet ratio index (APRI).

### 2.3. Bile Acid (BA) Quantification by Liquid Chromatography-Tandem Mass Spectrometry (LC-MS/MS)

BA concentrations were quantified by LC-MS/MS, as described previously [1, 32, 46, 48]. Briefly, a Waters ACQUITY ultraperformance liquid chromatography (UPLC) system (Waters, Milford, MA, USA) coupled to an Applied Biosystem 4000 Q TRAP® quadrupole linear ion trap hybrid mass spectrometer with an electrospray ionization (ESI) source (Applied Biosystems, MDS Sciex, Foster City, CA, USA) was used to perform the LC-MS/MS analysis. All chromatographic separations were performed with an ACQUITY UPLC® BEH C18 column (2.1 × 150 mm, 1.7 *μ*m) equipped with an ACQUITY UPLC C18 guard column (Waters, Milford, MA, USA).

### 2.4. Sample Preparation

Solid phase extraction was used to extract urine samples as mentioned previously [1, 32, 45, 54, 55]. 100 *μ*L of urine samples were spiked with 10 *μ*L of internal standard (IS), vortexed and loaded on to SupelcleanTM LC-18 SPE cartridges preconditioned with 4 mL MeOH, followed by 4 mL H2O. Loaded cartridges were then washed with 3 mL H2O and eluted with 4 mL MeOH. The eluates were evaporated under vacuum at room temperature and reconstituted in a 100 *μ*L of 50% MeOH solution. Ten microliters of reconstituted samples was injected for LC-MS/MS analysis.

### 2.5. Calculation of BA Indices

The BA profile in urine was characterized using BA “indices,” as we have described previously [1, 32, 46, 48].  Table 1 shows a summary of the BA indices used in the current study. BA indices describe the composition, hydrophilicity, formation of 12*α*-OH BA by CYP8B1, metabolism, and formation of secondary BA by intestinal bacteria. The composition indices were calculated as the ratio of the concentration of individual BA in all their forms (unamidated, amidated, unsulfated, and sulfated) to the total concentration of BA. Hydrophilicity indices include the percentages of the BA pool exist as mono-, di-, or tri-OH BA as well as the hydrophobicity index (HI) of the BA pool. The percentages of mono-OH BA (LCA), di-OH BA (UDCA, MDCA, HDCA, DCA, and CDCA), and tri-OH BA (CA, MCA, and HCA) were calculated as the ratio of the concentration of the sum of the respective BA in all their forms to the total concentration of BA. HI was calculated according to the Heuman index, which based on the relative contributions of the individual BA to the total BA pool and their His [56].

12*α*-OH BA are formed by CYP8B1 in the liver and include DCA, CA, Nor-DCA, and 3-dehydroCA. Therefore, CYP8B1 activity can be measured by the ratio of 12*α*-OH BA to the remaining of all other BA (non-12*α*-OH BA). Another marker for CYP8B1 is the ratio of CA to CDCA because CA is formed by the 12*α* hydroxylation of CDCA. In the same way, the ratio of 12*α*-OH (DCA, CA, Nor-DCA, and 3-dehydroCA in all their forms) to non-12*α*-OH (HDCA, CDCA, UDCA, LCA, MDCA, MCA, HCA, 12-oxo-CDCA, 6-oxo-LCA, 7-oxo-LCA, 12-oxo-LCA, isoLCA, and isoDCA in all their forms) was calculated.

BA are primarily metabolized by sulfation, glycine (G), and taurine (T) amidation in the liver. The percentage of sulfation of individual BA was calculated as the ratio of the concentration of sulfated BA, in both the unamidated and amidated forms, to the total concentration of individual BA in all of their forms (unamidated, amidated, unsulfated, and sulfated). The percentage of amidation of individual BA was calculated as the ratio of the concentration of amidated BA, in both the unsulfated and sulfated forms, to the total concentration of individual BA in all of their forms (unamidated, amidated, unsulfated, and sulfated). In addition, percentages of amidation were divided into the percentages of BA existing as taurine (T) or as glycine (G) amidates.

Primary BA are synthesized in the liver and secreted into the intestine via bile, where they are metabolized by intestinal bacteria into secondary BA. The ratio of primary (CA, CDCA, MCA, and HCA in all their forms) to secondary BA (DCA, LCA, UDCA, HDCA, MDCA, Nor-DCA, 12-oxo-CDCA, 3-dehydroCA, 6-oxo-LCA, 7-oxo-LCA, 12-oxo- LCA, isoLCA, and isoDCA in all their forms) was also calculated.

### 2.6. Statistical Analysis

To develop prognostic models, logistic regression model was used to predict the prognosis of hepatobiliary diseases in terms of developing disease-related complications. Models were constructed to predict (i) various individual complications and (ii) all complications combined (pooled) in the entire liver-patient population as well as in the individual disease subtype-populations (patient groups with specific disease subtypes). All statistical analyses were conducted using the Statistical Product and Service Solutions (SPSS) software, version 26 (IBM corporation, Armonk, NY, USA).

We developed models with six different sets of predictors: (i) BA variables only, (ii) Non-BA variables only, (iii) Mixed BA and non-BA variables, (iv) original model for end-stage liver disease (MELD), (v) MELD variable with coefficients from our data set, and (vi) original MELD modified with BA and/or non-BA variables.

Individual BA and/or non-BA variables were analyzed as possible predictors in a univariate logistic regression analysis. Significant variables (*P* value < 0.05) were selected from the univariate analysis to include in the multivariate analysis. The backward elimination method was used to avoid multicollinearity and retain the statistically significant variables with retention criteria during the multivariate analysis.

The estimated odds ratio (OR) of developing complications by BA and/or non-BA variables was obtained from the final multivariate logistic regression model for all subjects.

(1) $$\begin{array}{c} \log\left(\hat{\text{OR}}\right)=\log\left[\frac{\hat{P}}{1-\hat{P}}\right]=a+b_{1}x_{1}+\cdots +b_{k}x_{k}, \end{array}$$

where $\hat{P}\;$ is the probability of developing complications; *a* is the estimated intercept; and *b*_1_, ⋯, *b*_*k*_ represent the estimated regression coefficients for the variables *x*_1_, ⋯, *x*_*k*_ [57].

The final multivariate logistic regression model provides the associations between significant BA and/or non-BA variables and the odds of developing complications. We then computed the predicted probability, which transforms the estimated probabilities of complications to a scale of 0 to 1 using the following equation:

(2) $$\begin{array}{c} \hat{P}=\frac{\exp\left(\log\left(\hat{\text{OR}}\right)\right)}{\;1+\exp\left(\log\left(\hat{\text{OR}}\right)\right)}. \end{array}$$

Goodness-of-fit was assessed by using the Hosmer–Lemeshow (HL) test for logistic regression models. This test compares the observed number of individuals to the expected number of individuals in each pattern, which shows how well the data fits into the model [57]. In general, the HL test indicates a poor fit if the *P* value is less than 0.05.

We used akaike information criterion (AIC) for model comparisons among logistic regression models with different sets of predictors [58]. Minimizing AIC values represents a better goodness-of-fit [59]. The AIC values were calculated by

(3) $$\begin{array}{c} \text{AIC}=-2\ln\left(L\right)+2K, \end{array}$$

where *L* is the likelihood evaluated at the maximum likelihood estimate and *K* is the number of parameters in the models [60].

Bootstrapping was used to validate the models. Bootstrapping is a resampling technique used to estimate statistics on a population by sampling a data set with replacements [61]. The parameters included *P* value, bias, and standard error (SE) [62]. The bootstrapping estimate of bias indicated the difference between the estimates computed using the original sample and the mean of the bootstrap estimate. The SE represented the standard deviation of the estimator and reflects how far our sample estimate deviates from the actual parameters [63]. The range of regression coefficients (B) was defined as a 95% confidence interval of the bootstrap estimator. A bootstrap estimate of bias is the difference between the estimate calculated using the original sample and the mean of the bootstrap estimates. Acceptance criteria of *P* values were set at 0.05.

We also performed receiver operating characteristic curve (ROC) on the scores from multivariate logistic regression models to determine their optimal cut-off value in differentiating patients with or without ascites. The cut-off values with optimum specificity vs. sensitivity were selected, and the areas under the ROC curve (AUC) values were calculated. AUC of 0.9 or greater is rarely seen, AUC between 0.8 and 0.9 indicates excellent diagnostic accuracy, and any AUC over 0.7 may be considered clinically useful [54, 57, 64, 65].

The performance of the different models in predicting the occurrence of complications was compared using statistical outcomes from the HL test, AIC values, bootstrapping, and AUC values.

## 3. Results

### 3.1. Demographics

Table 2 shows a summary of the demographics of patients, who participated in this study. During the 7-year follow-up period, there were 257 patients with cholestatic liver diseases. The development of the following liver disease-related complications was monitored: ascites (62), bacterial peritonitis (2), encephalopathy (36), GI bleeding (18), hepatobiliary carcinoma (15), hepatorenal syndrome (1), jaundice (7), peripheral edema (63), and portal hypertension (106).

### 3.2. Univariate Logistic Regression Analysis for Ascites Prediction in the Entire Liver-Patient Population

Table 3 shows the results of univariate logistic regression analyses for ascites prediction by BA indices in the entire liver-patient population. The odds ratio (OR) quantifies the magnitude of the risk of developing ascites per one unit as well as 10% and 20% change of the normal values of BA indices. We found a correlation between the odds of developing ascites and many BA indices (*P* values < 0.05). Positive regression coefficient (*B*) values indicate that odds of developing ascites increase with increasing the values of BA indices, while negative coefficients imply the odds of developing ascites increase with decreasing the value of BA indices. For example, for every 20% increase in the % CDCA, the odds of developing ascites increased 1.4-fold (OR: 1.387; *P* value < 0.05). In contrast for every 20% increase in % MDCA, the odds of developing ascites decreased 0.774-fold (OR: 0.774; *P* value< 0.05).

We performed the same univariate logistic regression analysis for demographics and non-BA parameters as well (Table 4). For demographics, gender was the only statistically significant variable (*P* value < 0.05), with the odds of developing ascites being 1.3-fold higher in males than females. For non-BA parameters, increasing levels of creatinine, INR, protime, AST, bilirubin, AST/ALT, and MELD significantly increased the odds of developing ascites, whereas decreasing levels of albumin and ALT significantly increased the odds of developing ascites. For example, for every 20% increase in the INR, the odds of developing ascites increased 1.4-fold (OR: 1.391; *P* value < 0.05). In contrast, for every 20% increase in the albumin, the odds of developing ascites decreased 0.23-fold (OR: 0.231; *P* value < 0.05).

## 4. Multivariate Logistic Regression Analysis for Ascites Prediction in the Entire Liver-Patient Population

### 4.1. The BALDC Model

In multivariate logistic regression analysis, a backward elimination method was used to identify a statistically relevant BA variable from univariate analysis. The only BA variables retained in the multivariate model were % MDCA and % primary BA, which were independently predictive of developing ascites (Table 5(a)). The estimated odds ratio (OR) of developing ascites as a function of BA variables (BA-$\hat{\text{OR}}$) for individual patients were calculated using this equation:

(4) $$\begin{array}{c} \text{BALDC score}=\text{Log}\left(\text{BA}­\right)=-3.463-\left(2.452\times \%\text{MDCA}\right)+\left(0.045\times \%\text{PrimaryBA}\right). \end{array}$$

The predicted probability $\left(\hat{P}\right)$ of ascites as a function of BALDC (BA-$\hat{P}$) variables was then calculated using this equation:

(5) $$\begin{array}{c} \text{BA}­\left(\hat{P}\right)=\frac{\exp\left(\text{Log}\left(\text{BA}­\hat{\text{OR}}\right)\right)}{\;1+\exp\left(\text{Log}\left(\text{BA}­\hat{\text{OR}}\right)\right)}. \end{array}$$

**Figure 1:**
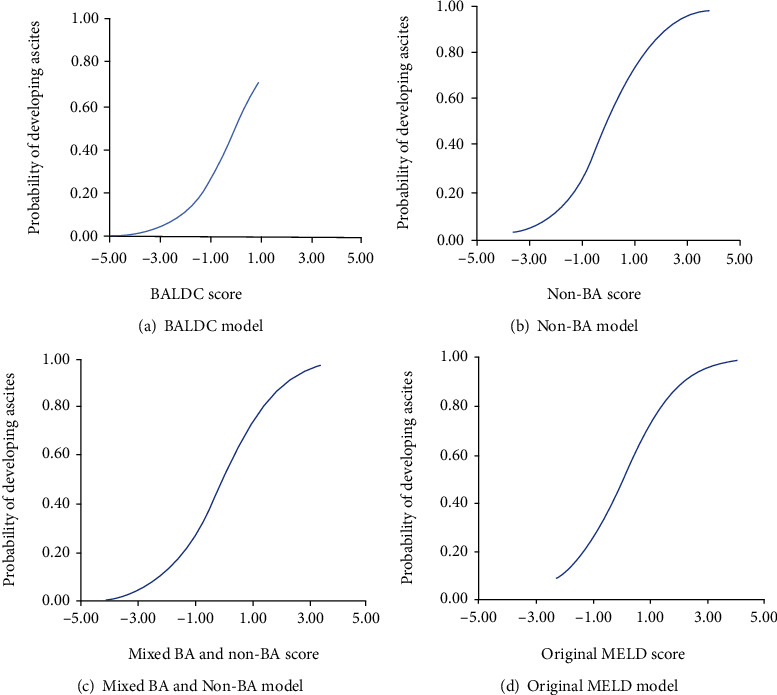
The relationship between the BALDC, non-BA, mixed BA and non-BA, and original MELD model scores and the probability of developing ascites.

Figure 1(a) shows the probability of developing ascites $\left(\text{BA}-\hat{P}\right)$ as predicted by the BALDC score.

For example, for a patient with a % MDCA of 1%, and % primary BA of 30%, the estimated odds ratio (BA-OR) of developing ascites by BA variables is as follows:

(6) $$\begin{array}{c} \text{BALDC score}=\text{Log}\left(\text{BA}­\right)=-3.463-\left(2.452\times 1\%\right)+\left(0.045\times 30\%\right)=-4.564. \end{array}$$

Then, the predicted probability of developing ascites (BA-$\hat{P}$) by BA variables can be calculated as

(7) $$\begin{array}{c} \text{BA}­\left(\hat{P}\right)=\frac{\exp\left(-4.565\right)}{\;1+\exp\left(-4.565\right)}=0.01. \end{array}$$

Furthermore, we tested the effect of the significant demographic variables from univariate analysis, i.e., gender, on this BADLC multivariate model. Gender was retained in the multivariate analysis but with no-minimal improvement of model validation and comparison criteria including bootstrapping, AIC, and ROC-AUC. Therefore, we did not include gender in the multivariate logistic regression model.

### 4.2. The Non-BA Model

We performed the same multivariate logistic regression analysis for non-BA parameters as well. Albumin level and MELD were the only significant predictive variables of developing ascites (Table 5(b)). The estimated odds ratio (OR) of developing ascites as a function of non-BA variables (non-BA-$\hat{\text{OR}}$) for individual patients was calculated from this equation:

(8) $$\begin{array}{c} \text{Non}-\text{BA score}=\text{Log}\left(\text{non}­\text{BA}­\hat{\text{OR}}\right)=0.947-\left(1.205\times \text{Albumin level }\left(\frac{\text{g}}{\text{dl}}\right)\right)+\left(0.189\times \text{MELD}\right). \end{array}$$

The predicted probability $\left(\hat{P}\right)$ of developing ascites as a function of non-BA $\left(\text{non}-\text{BA}-\hat{P}\right)$ variables was calculated using this equation:

(9) $$\begin{array}{c} \text{Non}­\text{BA}­\left(\hat{P}\right)=\frac{\exp\left(\log\text{Log}\left(\text{non}­\text{BA}­\hat{\text{OR}}\right)\right)}{\;1+\exp\left(\log\text{Log}\left(\text{non}­\text{BA}­\hat{\text{OR}}\right)\right)}. \end{array}$$

Figure 1(b) shows the probability of developing ascites as predicted by the non-BA score.

### 4.3. The Mixed BA and Non-BA Model

For mixed BA and non-BA variables, the variables retained in the multivariate model were % CDCA, primary/secondary BA, albumin level, and MELD which were independently predictive of developing ascites (Table 5(c)). The estimated odds ratio (OR) of developing ascites by mixed BA and non-BA for individual patients was calculated from this equation:

(10) $$\begin{array}{c} \text{Mixed BA and non}-\text{BA score}=\text{Log }\left(\text{mixed BA and non}­\text{BA}­\hat{\text{OR}}\right)=-0.275+\left(0.029\times \%\text{CDCA}\right)-\left(0.077\times \frac{\text{PrimaryBA}}{\text{SecondaryBA}}\right)-\left(1.143\times \text{Albumin level}\left(\frac{\text{g}}{\text{dl}}\right)\right)+\left(0.189\times \text{MELD}\right). \end{array}$$

The predicted probability $\left(\hat{P}\right)$ of developing ascites as a function of mixed BA and $\text{non}-\text{BA }\left(\text{mixed BA and non}-\text{BA}-\hat{P}\right)$ variables was calculated using this equation:

(11) $$\begin{array}{c} \text{Mixed BA and non}­\text{BA}­\left(\hat{\text{P}}\right)=\frac{\exp\left(\log\text{Log}\left(\text{mixed BA and non}­\text{BA}­\hat{\text{OR}}\right)\right)}{\begin{array}{c} \;\\ 1+\exp\left(\log\text{Log}\left(\text{mixed BA and non}­\text{BA}­\hat{\text{OR}}\right)\right) \end{array}} \end{array}$$

Figure 1(c) shows the probability of developing ascites as predicted by the mixed BA and non-BA score.

### 4.4. The Original MELD Model

We also performed the same multivariate logistic regression analysis for the MELD parameter (Table 5(d)). The estimated odds ratio (OR) of developing ascites as a function of original MELD variables for individual patients was calculated from this equation:

(12) $$\begin{array}{c} \text{Original MELD score}=\log\left(\text{MELD}-\hat{\text{OR}}\right)=-4.049+\left(0.276\times \text{MELD}\right). \end{array}$$

The predicted probability ($\hat{P}$) of developing ascites as a function of original MELD variables was calculated using this equation:

(13) $$\begin{array}{c} \text{MELD}­\left(\hat{\text{P}}\right)=\frac{\exp\left(\log\text{Log}\left(\text{MELD}\right)\right)}{\;1+\exp\left(\log\text{Log}\left(\text{MELD}\right)\right)}. \end{array}$$

Figure 1(d) shows the probability of developing ascites as predicted by the original MELD score.

### 4.5. Other Hybrid Models

In addition, we used the same methodology to develop other models (Supplementary Table S1) including (i) MELD variables with coefficients from our data set to create a model with the original MELD variables, but with model coefficients derived from our data set. In this model, creatinine and INR variables from the original MELD were not statistically significant. (ii) Original MELD modified with BA or non-BA variables at a time, to test if the performance of the original MELD could be improved by adding significant BA or non-BA parameters from the univariate analysis. Original MELD modified with BA variables only did not pass the HL test (*P* value < 0.05), while original MELD modified with non-BA variables only did improve the performance of the original MELD variables. However, this model has poor performance because of the low AUC (0.865) and high AIC (171) values compared to the mixed BA and non-BA model. (iii) Original MELD was modified with both BA and non-BA variables, to test if the performance of the original MELD could be improved by adding both significant BA and non-BA parameters from the univariate analysis. This model did not result in any improvement compared to the mixed BA and non-BA model (Table 5(c)). In this model's performance, AUC (0.875) and AIC (167) values were the same as the mixed BA and non-BA model. Since none of these models has improved the performance of our main models, we did not further evaluate any of these approaches.

### 4.6. Model Goodness-of-fit, Validation, and Performance

The Hosmer–Lemeshow (HL) test was used as one criteria to evaluate goodness-of-fit for all logistic regression models. The HL *P* values were 0.17 for BALDC, 0.23 for non-BA, and 0.11 for mixed BA and non-BA model. HL *P* values above 0.05 means that the observed and expected results were not significantly different, indicating the logistic regression of these models fit the data well. In contrast, for the original MELD model, the *P* value of the HL test was 0.029 (*P* value < 0.05), indicating the logistic regression of the original MELD model did not fit the data well (Table 6).

Table 6 also shows the akaike information criterion (AIC) for ascites prediction. AIC values were used to compare models with different error distribution. The AIC values for the BALDC, non-BA, mixed BA and non-BA, and original MELD models were 223.56, 170.81, 167.3, and 180.45. The BALDC model had a larger AIC value than the non-BA, mixed BA and non-BA, and original MELD models. This indicates that the logistic regression of the BALDC model did not fit the data well compared to the other candidate models.

Table 7 describes the bootstrapping validation for ascites prediction. Bootstrapping validation results for all four models indicated that the regression coefficients (*B*) were in the range of 95% confidence intervals, and *P* values were statistically significant for all covariates (*P* value < 0.05). Bias values were relatively small (-0.056 to 0.016), which means the estimates calculated using the original sample and the mean of the bootstrap estimate were not significantly different. In contrast, standard error (SE) and relative standard error (RSE) (0.02% to 296.3%) values of the bootstrapping analysis were relatively high, which may reflect our sample estimate derivates far from the actual parameter (Supplementary Figure S1).

**Figure 2:**
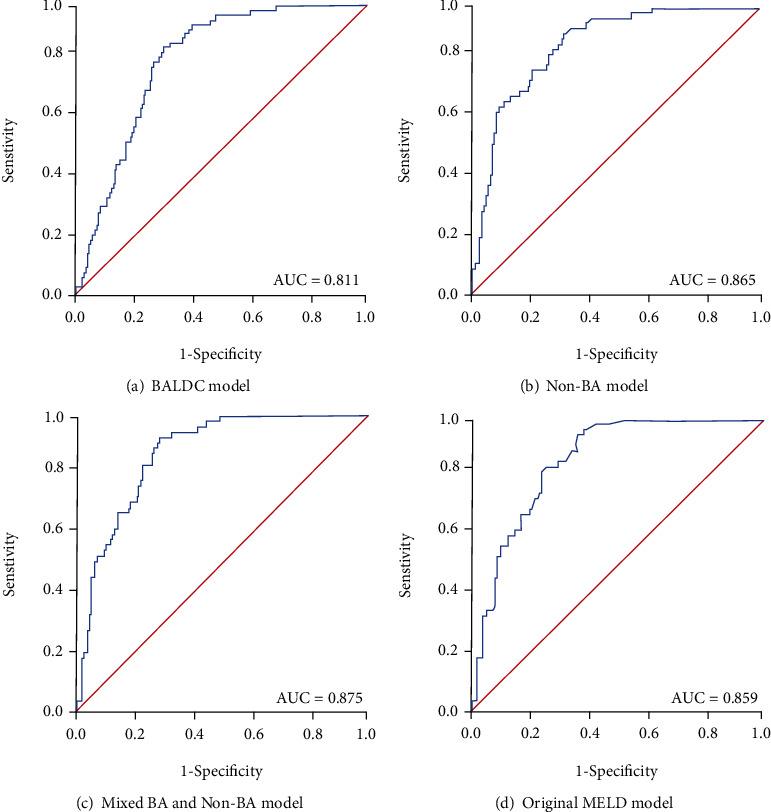
Receiver operating characteristic (ROC) curves of the BALDC, non-BA, mixed BA and non-BA, and original MELD models for ascites prediction. The area under the ROC curves (AUC) for (a) BALDC model, (b) non-BA model, (c) mixed BA and non-BA model, and (d) original MELD model for differentiating patients with ascites from patients without ascites.

Figure 2 shows the receiver operating characteristic (ROC) curves of all four models for ascites prediction. The area under the ROC curve for the BALDC, non-BA, mixed BA and non-BA, and original MELD was 0.81, 0.87, 0.88, and 0.86, respectively.

We also calculated the sensitivity (SEN), specificity (SPE), positive predictive value (PPV), and negative predicative values (NPV) from ROC analysis (Table 6). For instance, in the BALDC model, the sensitivity and specificity were 33.90% and 88.30% and the positive and negative predictive values were 48.80% and 80.20%.

Potential cut-off values of all 4 model scores to best differentiate patients with vs. without ascites were selected based on the optimum sensitivity vs. specificity from ROC analysis. The ROC-optimum cut-off values for BALDC, non-BA, mixed BA and non-BA models, and original MELD models for ascites prediction were -0.99, -1.18, -1.06, and -1.09, respectively (Table 6).

**Figure 3:**
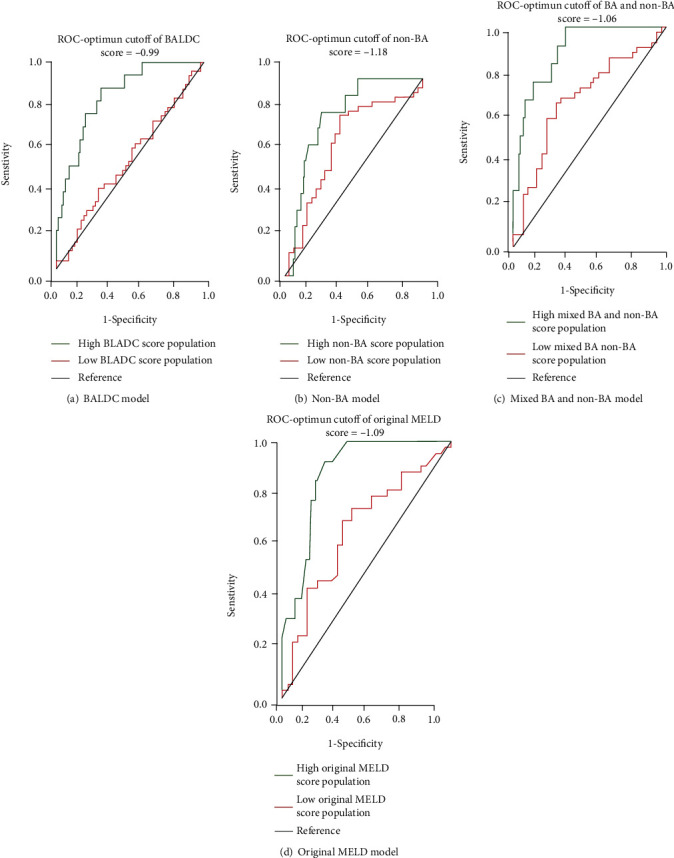
ROC analysis using optimum cut-off values in BALDC, non-BA, mixed BA and non-BA, and original MELD model scores.

Moreover, we tested if patient populations with scores below vs. higher than these optimum cut-off values can be distinguished using ROC analysis. The *P* value of AUCs was used to find statistically significant differences between the low- vs. high-score populations (Figure 3 and Table 8). The null hypothesis for *P* value of AUCs was AUC = 0.5.

### 4.7. Prediction for Other Complications

We also followed the same approach to predict other complications of liver diseases including bacterial peritonitis, encephalopathy, GI bleeding, hepatobiliary carcinoma, hepatorenal syndrome, and portal hypertension. Supplementary Table S2 shows the ROC analyses, *P* values of the bootstrapping, HL tests, and AICs for the BALDC models. Supplementary Table S3–5 show similar results for non-BA, mixed BA and non-BA, and original MELD models.

## 5. Discussion

In this study, we have examined the ability of BA indices to predict complications in patients with liver diseases. Logistic regression model was used to predict the prognosis of hepatobiliary diseases in terms of developing disease-related complications. In addition to the BALDC model, we have developed (i) non-BA, (ii) mixed BA, and non-BA variables to compare with the BA-only and non-BA-only models. (iii) MELD variables with coefficients from our data set were used to create a model with the original MELD variables, but with model coefficients derived from our data set. (iv) Original MELD was modified with BA and/or non-BA variables, to test if the performance of original MELD can be improved by adding significant BA and non-BA parameters from the univariate analysis. First, individual BA and non-BA variables were analyzed as possible predictors of developing ascites in a univariate logistic regression analysis. Then, multivariate models were built using backward elimination regression, where only the most significant variables from the univariate regression were retained.

The final multivariate logistic regression models were then validated using bootstrapping method. Goodness-of-fit criteria also included the HL test, the AIC, and multivariate parameters from the receiver operating characteristic analyses.

From univariate logistic regression analysis, total UDCA, total CA, total MCA, % CDCA, % sulfation, total Mono-OH, % T-amidation, % tri-OH, % non-12*α*-OH, and % primary BA significantly increased the odds of having ascites, whereas total DCA, total HDCA, % LCA, % G-amidation, % mono-OH, and % secondary BA decreased the odds of having ascites (Table 3).

For demographics, univariate logistic regression analysis showed that the odds of having ascites was significantly 1.3-fold higher in males than females. For non-BA parameters, creatinine, INR, protime AST, bilirubin, AST/ALT, and MELD increased the odds of having ascites, whereas albumin and ALT decreased the odds of having ascites (Table 4).

Using multivariate logistic regression analysis, we have constructed these final models for ascites prediction:

- The BA variables (BA-$\hat{\text{OR}}$) model for ascites prediction:

(14) $$\begin{array}{c} \text{BALDC score}=\text{Log}\left(\text{BA}­\hat{\text{OR}}\right)=-3.463-\left(2.452\times \%\text{MDCA}\right)+\left(0.045\times \%\text{PrimaryBA}\right) \end{array}$$

(ii) The non-BA variables (non-BA-$\hat{\text{OR}}$) model for ascites prediction:

(15) $$\begin{array}{c} \text{Non}-\text{BA score}=\text{Log}\left(\text{non}-\text{BA}­\hat{\text{OR}}\right)=0.947-\left(1.205\times \text{Albumin level}\left(\frac{\text{g}}{\text{dl}}\right)\right)+\left(0.189\times \text{MELD}\right) \end{array}$$

(iii) The original MELD variable (MELD-$\hat{\text{OR}}$) model for ascites prediction:

(16) $$\begin{array}{c} \text{Original MELD score}=\text{Log}\left(\text{MELD}­\hat{\text{OR}}\right)=-4.049+\left(0.276\times \text{MELD}\right) \end{array}$$

(iv) The mixed BA and non-BA variables (mixed BA and non-BA-$\hat{\text{OR}}$) model for ascites prediction:

(17) $$\begin{array}{c} \text{Mixed BA and non}-\text{BA score}=\text{Log }\left(\text{mixed BA and non}-\text{BA}-\hat{\text{OR}}\right)=-0.275+\left(0.029\times \%\text{CDCA}\right)-\left(0.077\times \frac{\text{PrimaryBA}}{\text{SecondaryBA}}\right)-\left(1.143\times \text{Albumin}\left(\frac{\text{g}}{\text{dl}}\right)\right)+\left(0.189\times \text{MELD}\right) \end{array}$$

Gender was the only significant demographic variable in univariate logistic regression analysis for all models (Table 4). However, it was not included in these models because it resulted in but with no-minimal improvement of model validation criteria including bootstrapping, AIC, and ROC-AUC. Therefore, we did not include gender in the multivariate logistic regression model.

Cholestatic diseases are associated with impaired bile flow to the intestine, which is expected to translate into reduced transformation of primary BA into secondary BA by intestinal bacteria. Therefore, an accumulation of primary and a decrease in secondary BA in the blood may indicate further impairment in bile flow and existing liver disease [1, 66–69]. This was in agreement with the BALDC model, where increasing % primary BA and decreasing % MDCA (a secondary BA) were the final significant predictors of liver disease prognosis. Furthermore, we have previously demonstrated survival model development for death prediction using cox regression analyses. The same results have shown in their BA model, where increased % CDCA and % Tri-OH BA (both are primary BA) were the significant predictors of liver disease prognosis into death.

As shown in Figure 1, the probability of developing ascites increased as a function of BALDC, non-BA, mixed BA and non-BA, and original MELD scores. In general, logistic regression analysis produces an S-shaped curve, when predicated probability is plotted against the explanatory score [70]. All four models produced such S-shaped curves except for the BALDC score. This is expected in the absence of extreme values of BALDC scores from our data set. However, with more subject enrollment in the future, more extreme BALDC score values; therefore, S-curve shapes are expected.

Hosmer–Lemeshow (HL) test was one of the criteria to evaluate the goodness-of-fit for logistic regression models. The HL test results supported the validity of the BALDC, non-BA, and mixed BA, and non-BA models (*P* value > 0.05), but not the original MELD model (Table 6). The original MELD model was the only model with *P* value < 0.05, which indicates the expected and observed results were significantly different. As an alternative, we considered a probit regression analysis to model the original MELD (data not shown). Based on our finding, the MELD with probit model showed a better performance compared to the logistic regression model; however, it was not fitted well in BA and non-BA models. Therefore, we use the logistic regression model for the entire analyses.

We also used akaike information criterion (AIC) to compare the estimated out-of-sample prediction error from multivariate logistic regression models. Smaller AIC values represent a better goodness-of-fit in model performance [59]. The AIC values of the BALDC, non-BA, and original MELD models were 233.56, 170.81, and 180.45, which were higher than the AIC value of the mixed BA and non-BA model (167.3) (Table 6).

Models were validated using the bootstrapping method (Table 7). Bootstrapping is a resampling technique used to estimate statistics on a population by sampling a data set with replacement [61]. Random samples were taken one at a time, with replacement from our data set to create a series of 1000 new data sets. Statistics were calculated by comparing these data sets. In the BALDC model, the relative standard error was relatively large because the model parameter (% MDCA) has a high relative standard error (Supplementary Figure S1). This could be due to the fact that % MDCA was not normally distributed in the original data set and because the sample size was relatively small [71]. Despite the high relative standard error, the BALDC model could be considered to pass the bootstrapping validation given the relatively small sample size of our study. Overall, the bootstrapping validation results supported the validity of the BALDC, non-BA, mixed BA and non-BA, and original MELD models for ascites prediction.

ROC analysis was used to compare the models for their accuracy to predict liver patient prognosis into complications such as ascites. The higher the area under the ROC curve, the greater the overall accuracy of the marker in distinguishing between groups. For prognostic models, AUC of 0.9 or greater is rarely seen. AUC between 0.8 and 0.9 indicates excellent accuracy. And any AUC over 0.7 may be considered clinically useful [72–74]. Therefore, all four models show high accuracy for ascites prediction.

ROC analysis was also performed to test sensitivity, specificity, and positive and negative predictive values (Table 6). The sensitivity is the proportion of true positive patients (patients who were predicted to have ascites and actually did have ascites) to the actual positive patient population (total number of patients who actually did have ascites). The specificity is the proportion of true negative patients (patients who were predicted not to have ascites and actually did not have ascites) to the actual negative patient population (total number of patients who actually did not have ascites). The positive predictive value is the proportion of true positive patients to the total number of predicted positive patients. The negative predictive value is the proportion of true negative patients to the total number of predicted negative patients. The high sensitivity and specificity correspond to the high positive and negative predictive values, and vice versa. Predictive values are more commonly used than sensitivity and specificity in clinical studies [70]. The higher positive and negative predictive values are preferred when comparing model performance. Based on that, we compared positive and negative predictive values for all four models. The non-BA model has higher positive and negative predictive values than other models. In addition, the mixed BA and non-BA model also has high predictive values closed to the non-BA model. Therefore, both non-BA and mixed BA and non-BA models show better model performance than others.

Moreover, ROC analysis was used to determine potential cut-off values which quantify the normal range of biomarkers. The selection of optimum cut-off values is a tradeoff between sensitivity vs. specificity, where lower cut-off values are associated with higher sensitivity but lower specificity, and vice versa. Scores for the BALDC, non-BA, mixed BA and non-BA, and original MELD models were identified as cut-off values with optimum sensitivity vs. specificity, which were -0.99, -1.18, -1.06, and -1.09, respectively (Table 6). For example, a BALDC score of -0.99 was considered an optimum cut-off value in differentiating patients with vs. without ascites because it maintained a balance between sensitivity (74%) vs. specificity (74%).

These ROC optimum cut-off values were used to split the overall patient population into two populations for every model. One population contained patients with model scores higher than the cut-off score and the other contained patients with model scores lower than the cut-off score. The *P* value of AUCs from the two populations for every model was then used to find statistically significant differences (Figure 3 and Table 8). The *P* value of AUCs is smaller than 0.05 and lead to the rejection of the null hypothesis, indicating AUCs are above the reference line (AUC = 0.5), and vice versa. Only ROC-optimum cut-offs for the BALDC score (-0.99) resulted in statistically significant different AUCs based on their *P* values; therefore, they were able to distinguish high- vs. low-score patient populations.

In addition to ascites, we attempted to develop similar models for the prediction of other common liver disease complications including bacterial peritonitis, encephalopathy, GI bleeding, hepatobiliary carcinoma, hepatorenal syndrome, and portal hypertension (Supplementary Table S3–5). None of these complications were as accurately predicted as ascites by any of the BALDC and non-BA models. In general, models for the prediction of other complications had lower sensitivity, lower specificity, lower AUC values, and higher AIC values. This could be due to the fact that other complications were less common than ascites (except for portal hypertension) in our study. Overall, improving prediction accuracy would require an increase in the study population to predict all these other complications.

## 6. Conclusions

We have developed and validated a prognosis model based on BA indices to predict the development of liver disease complications such as ascites. Other models, including non-BA, mixed BA and non-BA, and original MELD models, were also developed to compare their performance with our BALDC model. Overall, the mixed BA and non-BA model was the most accurate based on AIC and ROC analyses. The mixed BA and non-BA had lower AIC values indicating a smaller error of distribution and a better trade-off between goodness-of-fit vs. degrees of freedom (Table 6). Moreover, the mixed BA and non-BA model had the highest AUC values indicating higher accuracy than other models (Figure 2). Therefore, the mixed BA and non-BA model could be used to predict the development of ascites in patients diagnosed with liver-disease at early stages of intervention, such as liver transplantation. This will assist in supply allocation and physician decisions when treating liver diseases.

## Figures and Tables

**Table 1:** List of BA indices.

Composition	Hepatic metabolism	Hydrophilicity	CYP8B1 activity	Intestinal contribution
Concentration of individual BA	Total sulfated	Total mono-OH	Total 12*α*-OH	Total primary
% of individual BA	Total G-amidated	Total Di-OH	Total non-12*α*-OH	Total secondary
	Total T-amidated	Total tri-OH	12*α*-OH/non12*α*-OH	Primary/secondary
	% Sulfation	% mono-OH	CA/CDCA	% primary
	% Amidation	% di-OH	% 12*α*-OH	% secondary
	% G-amidation	% tri-OH	% non-12*α*-OH	
	% T-amidation	HI		

BA: bile acids; G: glycine; T: taurine; CDCA: chenodeoxycholic acid; CA: cholic acid.

**Table 2:** Demographics.

	Patients
*n*	257
*Gender*	
Male	136
Female	121
*Age* (*yr*)	
Mean ± SEM	52.2 ± 0.71
*Body mass index*	
Mean ± SEM	30.7 ± 0.45
*Race*	
White	217
Black	11
Asian	7
Hispanic	4
Others	18
*Liver disease complications*	
Ascites	62
Bacterial peritonitis	2
Encephalopathy	36
GI bleeding	18
Hepatobiliary carcinoma	15
Hepatorenal syndrome	1
Jaundice	7
Peripheral edema	63
Portal hypertension	106

**Table 3:** Univariate logistic regression analyses for the prediction of developing ascites in the entire liver-patient population based on BA indices.

BA (*μ*M)/BA indices	B-value (regression coefficient)	*P* value	Odds ratio (OR): Exp (*B*)		
			1 unit change	10% change	20% change
Total BA	0.002	0.059	1.002	1.010	1.020
Total LCA	0.024	0.275	1.024	1.007	1.013
Total UDCA	0.001	0.538	1.001	1.002	1.004
Total CDCA	0.009	0.002	1.009	1.017	1.034
Total DCA	-0.001	0.871	0.999	0.999	0.999
Total HDCA	-20.099	1.000	0.001	0.980	0.961
Total MDCA	-20.104	0.999	0.001	0.923	0.851
Total CA	0.052	0.007	1.053	1.013	1.027
Total MCA	0.008	0.528	1.008	1.002	1.005
Total HCA	0.407	0.012	1.502	1.007	1.015
% LCA	-0.071	0.004	0.931	0.936	0.877
% UDCA	-0.049	0.001	0.952	0.892	0.795
% CDCA	0.048	0.001	1.049	1.178	1.387
% DCA	-0.061	0.001	0.941	0.908	0.825
% HDCA	-6.66	0.108	0.001	0.980	0.960
% MDCA	-3.281	0.003	0.038	0.880	0.774
% CA	0.065	0.005	1.067	1.040	1.081
% MCA	-0.007	0.713	0.993	0.996	0.991
% HCA	-0.671	0.001	0.511	0.977	0.954
Total Unamidated	0.016	0.076	1.016	1.009	1.017
Total G-amidated	0.002	0.103	1.002	1.008	1.017
Total T-amidated	0.019	0.016	1.019	1.011	1.021
% Amidation	0.041	0.017	1.042	1.433	2.054
% G-amidation	-0.004	0.665	0.996	0.970	0.940
% T-amidation	0.037	0.002	1.038	1.039	1.080
Total Unsulfated	0.061	0.076	1.016	1.009	1.017
Total sulfated	0.002	0.061	1.002	1.009	1.018
% Sulfation	0.012	0.338	1.012	1.106	1.224
Total mono-OH	0.024	0.275	1.024	1.007	1.013
Total Di-OH	0.002	0.074	1.002	1.008	1.017
Total tri-OH	0.018	0.029	1.018	1.010	1.021
% mono-OH	-0.071	0.004	0.931	0.936	0.877
% Di-OH	0.018	0.095	1.018	1.142	1.304
% tri-OH	0.021	0.108	1.021	1.027	1.055
Total 12*α*-OH	0.008	0.162	1.008	1.007	1.014
Total non-12*α*-OH	0.002	0.068	1.002	1.008	1.017
12*α*-OH/non12*α*-OH	-0.787	0.114	0.455	0.974	0.948
CA/CDCA	-0.997	0.159	0.369	0.974	0.949
% 12*α*-OH	-0.033	0.014	0.968	0.928	0.861
% non-12*α*-OH	0.033	0.014	1.034	1.291	1.666
Total primary	0.007	0.003	1.007	1.017	1.034
Total secondary	0.001	0.543	1.001	1.003	1.005
Primary/secondary	0.09	0.001	1.094	1.020	1.041
% primary	0.049	0.001	1.050	1.258	1.582
% secondary	-0.049	0.001	0.952	0.770	0.594
HI	0.074	0.012	1.077	0.999	0.998

BA concentrations are in (*μ*M), while BA indices are in percentage.

**Table 4:** Univariate logistic regression analyses for the prediction of developing ascites in the entire liver-patient population based on demographics and non-BA parameters.

Demographics and non-BA parameters	*B* value	*P* value	Odds ratio (OR): Exp (*B*)		
			1 unit change	10% change	20% change
Age (yr)	0.012	0.366	1.012	1.000	1.001
BMI	-0.008	0.685	0.992	1.000	0.999
Gender	1.291	0.001	3.636	NA	NA
Race	∗	0.258	∗	∗	∗
Creatinine (mg/dL)	0.048	0.601	1.049	1.005	1.010
Albumin (g/dL)	-1.980	0.001	0.138	0.481	0.231
INR	1.529	0.001	4.614	1.180	1.391
Protime (sec)	0.133	0.001	1.142	1.156	1.337
AST (U/L)	0.003	0.168	1.003	1.017	1.034
ALT (U/L)	-0.004	0.257	0.996	0.977	0.955
Bilirubin (mg/dL)	0.536	0.001	1.709	1.069	1.142
AST/ALT	1.895	0.001	6.653	1.246	1.552
MELD	0.276	0.001	1.318	1.281	1.642

*B* value: regression coefficient; ^∗^Race is a categorical variable which contains five race groups. There are five values for *B* value and HR, one for each race group, which are not shown, because was not statistically significant in univariate logistic regression analysis; BMI: body mass index; INR: international normalized ratio; AST: aspartate transaminase; ALT: alanine transaminase; MELD: model for end-stage liver disease. NA: not applicable.

**Table:** (a) BALDC model

BA parameters	*B* value	Standard error	*P* value	Odds ratio (OR): Exp (*B*)		
				1-unit	10%	20%
Intercept	-3.463	—	0.001	0.031	—	—
% MDCA	-2.452	1.112%	0.027	0.086	0.909	0.826
% Primary BA	0.045	0.008%	0.001	1.046	1.234	1.524

Using the regression coefficients (*B*) from this table, the estimated (OR) of developing ascites by the BALDC model is BALDC score = Log (BA­OR) = −3.463 − (2.452 × %MDCA) + (0.045 × %primary BA).

**Table:** (b) Non-BA model

Non-BA parameters	*B* value	Standard error	*P* value	Odds ratio (OR): Exp (*B*)		
				1-unit	10%	20%
Intercept	0.947	—	0.560	2.577	—	—
MELD	0.189	0.050	0.001	1.208	1.185	1.404
Albumin level	-1.205	0.387	0.002	0.300	0.640	0.410

Using the regression coefficients (*B*) from this table, the estimated (OR) of developing ascites by the non-BA model is non‐BA score = Log (Non‐BA‐OR) = 0.947 + (0.189 × MELD) − (1.205 × albumin level).

**Table:** (c) Mixed BA and Non-BA model

Mixed BA and non-BA parameters	*B* value	Standard error	*P* value	Odds ratio (OR): Exp (*B*)		
				1-unit	10%	20%
Intercept	-0.275	1.768	0.894	0.79	—	—
% CDCA	0.029	0.012%	0.014	1.029	1.104	1.218
Primary BA/secondary BA	-0.077	0.032	0.015	0.926	0.983	0.967
Albumin level	-1.143	0.407	0.004	0.319	0.655	0.429
MELD	0.189	0.053	0.001	1.208	1.185	1.404

Using the regression coefficients (*B*) from this table, the estimated (OR) of developing ascites by the mixed BA and non-BA model is mixed BA and non − BA score = Log (BA‐OR) = −0.275 + (0.029 × %CDCA) − (0.077 × primary BA/secondary BA) − (1.143 × albumin level) + (0.189 × MELD).

**Table:** (d) Original MELD model

MELD parameters	*B* value	Standard error	*P* value	Odds ratio (OR): Exp (*B*)		
				1-unit	10%	20%
Intercept	-4.049	0.554	0.001	1.317	—	—
MELD	0.276	0.045	0.001	0.017	0.026	0.001

Using the regression coefficients (*B*) from this table, the estimated (OR) of developing ascites by the original MELD model is original MELD score = Log (MELD − OR) = −4.049 + (0.276 × MELD).

**Table:** (a) BALDC model

ROC analysis					HL (*P* value)	AIC value
SEN	SPE	PPV	NPV	Cutoff value (SEN, SPE)		
33.90%	88.30%	48.80%	80.20%	-0.99 (74%, 74%)	0.168	223.56

**Table:** (b) Non-BA model

ROC analysis					HL(*P* value)	AIC value
SEN	SPE	PPV	NPV	Cutoff value (SEN, SPE)		
56.40%	91.50%	72.10%	84.30%	-1.18 (78%, 78%)	0.228	170.81

**Table:** (c) Mixed BA and Non-BA model

ROC analysis					HL(*P* value)	AIC value
SEN	SPE	PPV	NPV	Cutoff value (SEN, SPE)		
54.50%	90.10%	68.2%	83.60%	-1.06 (78%, 78%)	0.11	167.3

**Table:** (d) Original MELD model

ROC analysis					HL(*P* value)	AIC value
SEN	SPE	PPV	NPV	Cutoff value (SEN, SPE)		
45.50%	91.50%	67.60%	81.30%	-1.09 (76%, 76%)	0.029	180.45

SEN: sensitivity; SPE: specificity; PPV: positive predictive value; NPV: negative predictive value; *P* value is for the Hosmer-Lemeshow test (HL); AIC: akaike information criterion.

**Table 7:** Bootstrapping validation for ascites predication models.

Variables	*B* value	Bias	SE	RSE	*P* value	95% CI	
						Lower	Upper
*BALDC model*							
Intercept	-3.463	-0.049	0.548	—	0.001	-4.666	-2.445
% MDCA	-2.452	-0.192	0.948%	296.3%	0.002	-4.823	-1.148
% PrimaryBA	0.045	-0.049	0.008%	0.02%%	0.001	0.032	0.061
*Non-BA model*							
Intercept	0.947	-0.056	1.702	—	0.554	-2.606	4.139
MELD	0.189	0.009	0.062	0.59%	0.001	0.086	0.325
Albumin_level	-1.205	-0.014	0.389	11.21%	0.001	-2.028	-0.490
*Mixed BA and non-BA model*							
Intercept	-0.236	-0.052	2.029	—	0.897	-4.572	3.484
% CDCA	0.029	-0.002	0.013%	0.03%	0.013	-0.001	0.052
Primary/secondary BA	-0.077	0.012	0.055	1.58%	0.028	-0.164	0.053
Albumin (g/dL)	-1.158	-0.023	0.46	13.26%%	0.005	-2.108	-0.219
MELD	0.189	0.016	0.066	0.63%	0.003	0.087	0.341
*Original MELD model*							
Intercept	-4.049	-0.098	0.658	—	0.001	0.183	0.411
MELD	0.276	0.007	0.061	0.59%.	0.001	-5.573	-2.996

*B* value: regression coefficient; SE: standard error; RSE: relative standard error; CI: confidence interval.

**Table 8:** ROC analysis using optimum cut-off values.

Cutoff	AUC	*P* value	SE	95% CI	
				Lower	Upper
*BALDC score*				
High BALDC score < −0.99	0.842	0.00	0.05	0.752	0.932
Low BALDC score ≥ −0.99	0.527	0.65	0.06	0.41	0.644
*Non-BA score*				
High non‐BA score < −1.18	0.806	0.00	0.05	0.707	0.905
Low non‐BA score ≥ −1.18	0.670	0.01	0.07	0.538	0.801
*Mixed BA and non-BA score*				
High BA and non‐BA score < −1.06	0.895	0.00	0.04	0.821	0.970
Low BA and non‐BA score ≥ −1.06	0.672	0.01	0.06	0.546	0.797
*Original MELD score*				
High original MELD score < −1.09	0.879	0.00	0.04	0.809	0.949
Low original MELD score ≥ −1.09	0.657	0.01	0.06	0.532	0.782

AUC: area under the ROC curve; SE: standard error; CI: confidence interval.

## Data Availability

Supplementary data are included and submitted with the article

## Abbreviations

AIC: Akaike information criterion
ALT: Alanine transaminase
AST: Aspartate transaminase
AUC: Area under the ROC curve
BA: Bile acids
BALDC: Bile-acid liver disease complications
B-value: Regression coefficient
CA: Cholic acid
CDCA: Chenodeoxycholic acid
CTP: Child-turcotte-pugh
CYP8B1: Cytochrome p450 family 8 subfamily B member 1
DCA: Deoxycholic acid
HDCA: Hyodeoxycholic acid
HI: Hydrophobicity index
HL: Hosmer–lemeshow
INR: International normalized ratio
IS: Internal standard
LCA: Lithocholic acid
LC-MS/MS: Liquid chromatography-tandem mass spectrometry
MCA: Muricholic acid
MDCA: Murideoxycholic acid
MELD: Model for end-stage liver disease
MeOH: Methanol
OH: Hydroxyl group
OR: Odds ratio
ROC: Receiver operating characteristics
SE: Standard error
UDCA: Ursodeoxycholic acid
UPLC: Ultra-performance liquid chromatography

## Supplementary Materials

Supplementary Materials Supplementary Figure S1: histograms for the BALDC, non-BA, mixed BA and non-BA, and original MELD model's variables. Std. Dev represents the standard deviation of each variable. *N* is the population of each variable. SE is the standard error of each variable. RSE represents the relative standard error of each variable. Supplementary Table S2: prediction of other liver disease complications using BALDC models. Supplementary Table S3: prediction of other liver disease complications using non-BA models. Supplementary Table S4: prediction of other liver disease complications using mixed BA and non-BA models. Supplementary Table S5: prediction of other liver disease complications using original MELD models.
