# Supplementary material for: Urinary BA Indices as Prognostic Biomarkers for Complications Associated with Liver Diseases
Source: Int J Hepatol. 2022 Mar 30;2022:5473752. doi: 10.1155/2022/5473752 (PMC8986411; doi:10.1155/2022/5473752)
Supplement: Supplementary Materials — Supplementary Figure S1: histograms for the BALDC, non-BA, mixed BA and non-BA, and original MELD model's variables. Std. Dev represents the standard deviation of each variable. N is the population of each variable. SE is the standard error of each variable. RSE represents the relative standard error of each variable. Supplementary Table S2: prediction of other liver disease complications using BALDC models. Supplementary Table S3: prediction of other liver disease complications using non-BA models. Supplementary Table S4: prediction of other liver disease complications using mixed BA and non-BA models. Supplementary Table S5: prediction of other liver disease complications using original MELD models. [file 5473752.f1.docx]

**Supplementary Materials**

**Supplementary Figure S1. Histograms for the BALDC, non-BA, mixed BA and non-BA, and original MELD model’s variables.** Std. Dev represents the standard deviation of each variable. N is the population of each variable. S.E is the standard error of each variable. RSE represents the relative standard error of each variable.

**Supplementary Figure S1. Histograms for the BALDC, non-BA, mixed BA and non-BA, and original MELD model’s variables**

1. **BALDC model’s variables**

1. **Non-BA model’s variables**

1. **Mixed BA and non-BA model’s variables**

**(d) Original MELD model**

**Supplementary Table S1. Other models for ascites prediction**

| Other Models | Logistic(P) | Bootstrapping(P) | HL(P) | AUC | AIC value |
| --- | --- | --- | --- | --- | --- |
| **MELD variables with coefficients from our data set** | | | | | |
| Creatinine | 0.739 | NA | NA | NA | NA |
| INR | 0.155 | NA |  |  |  |
| Bilirubin | 0.001 | NA |  |  |  |
| **Original MELD modified with BA variables** | | | | |  |
| MELD | 0.001 | 0.002 | 0.037 | 0.859 | 171 |
| %PrimaryBA | 0.009 | 0.005 |  |  |  |
| **Original MELD modified with non-BA variables** | | | | | |
| MELD | 0.001 | 0.001 | 0.228 | 0.865 | 171 |
| Albumin level | 0.002 | 0.001 |  |  |  |
| **Original MELD modified with BA and non-BA variables** | | | | | |
| %CDCA | 0.014 | 0.013 | 0.11 | 0.875 | 167 |
| Primary/SecondaryBA | 0.015 | 0.028 |  |  |  |
| Albumin level | 0.005 | 0.005 |  |  |  |
| MELD | 0.001 | 0.003 |  |  |  |

(P) is P-value; NA: Not applicable. Bootstrapping was not performed because P-values of model parameters were not significant (P-value > 0.05). HL: Hosmer–Lemeshow test; AUC: Area under the ROC curve; AIC: Akaike information criterion.

|  | **ROC Analysis** | | | | |  |  |  |
| --- | --- | --- | --- | --- | --- | --- | --- | --- |
| **Other complications** | **SEN** | **SPE** | **PPV** | **NPV** | **AUC** | **B(P)** | **HL(P)** | **AIC value** |
| Bacterial peritonitis | 0% | 100% | 0% | 99.2% | 0.952 | 0.001 | 0.967 | 22.39 |
| Encephalopathy | 2.8% | 98.1% | 20.0% | 85.7% | 0.777 | 0.001 | 0.744 | 177.75 |
| GI bleeding | 0% | 100% | 0% | 92.8% | 0.791 | 0.001 | 0.027 | 112.81 |
| Hepatobiliary  carcinoma | 0% | 100% | 0% | 94% | 0.745 | 0.001 | 0.714 | 104.52 |
| Hepatorenal. syndrome | NA | NA | NA | NA | NA | NA | NA | NA |
| Portal hypertension | 63.2% | 82.6% | 72.8% | 75.3% | 0.813 | 0.001 | 0.480 | 266.10 |

**Supplementary Table S2. Prediction of other liver disease complications using BALDC models.**

SEN: Sensitivity; SPE: Specificity; PPV: Positive predictive value; NPV: Negative predictive value; HL: Hosmer–Lemeshow test; AUC: Area under the ROC curve. B(p) is the P value for Bootstrapping method. AIC: Akaike information criterion; NA: Not applicable.

**Supplementary Table S3. Prediction of other liver disease complications using non-BA models.**

|  | **ROC Analysis** | | | | |  |  |  |
| --- | --- | --- | --- | --- | --- | --- | --- | --- |
| **Other complications** | **SEN** | **SPE** | **PPV** | **NPV** | **AUC** | **B(P)** | **HL(P)** | **AIC value** |
| Bacterial peritonitis | NA | NA | NA | NA | NA | NA | NA | NA |
| Encephalopathy | 24.2% | 97.0% | 61.5% | 86.4% | 0.829 | 0.001 | 0.140 | 145.28 |
| GI bleeding | 0.0% | 100.0% | 0.0% | 92.9% | 0.762 | 0.001 | 0.588 | 105.72 |
| Hepatobiliary  carcinoma | NA | NA | NA | NA | NA | NA | NA | NA |
| Hepatorenal. syndrome | NA | NA | NA | NA | NA | NA | NA | NA |
| Portal hypertension | 67.4% | 82.2% | 78.0% | 72.8% | 0.818 | 0.005 | 0.251 | 213.17 |

SEN: Sensitivity; SPE: Specificity; PPV: Positive predictive value; NPV: Negative predictive value; HL: Hosmer–Lemeshow test; AUC: Area under the ROC curve. B(p) is the P value for Bootstrapping method. AIC: Akaike information criterion; NA: Not applicable.

**Supplementary Table S4. Prediction of other liver disease complications using mixed BA and non-BA models.**

|  | **ROC Analysis** | | | | |  |  |  |
| --- | --- | --- | --- | --- | --- | --- | --- | --- |
| **Other complications** | **SEN** | **SPE** | **PPV** | **NPV** | **AUC** | **B(P)** | **HL(P)** | **AIC value** |
| Bacterial peritonitis | 0.0% | 100.0% | 0.0% | 99.2% | 0.952 | 0.004 | 0.967 | 22.39 |
| Encephalopathy | 24.2% | 86.4% | 61.5% | 86.4% | 0.829 | 0.001 | 0.14 | 145.28 |
| GI bleeding | 0.0% | 100% | 0.0% | 92.8% | 0.809 | 0.008 | 0.886 | 111.72 |
| Hepatobiliary  carcinoma | 0.0% | 100% | 0.0% | 94.0% | 0.717 | 0.001 | 0.703 | 107.07 |
| Hepatorenal. syndrome | NA | NA | NA | NA | NA | NA | NA | NA |
| Portal hypertension | 67.7% | 87.4% | 80.7% | 77.6% | 0.858 | 0.006 | 0.09 | 223.88 |

SEN: Sensitivity; SPE: Specificity; PPV: Positive predictive value; NPV: Negative predictive value; HL: Hosmer–Lemeshow test; AUC: Area under the ROC curve. B(p) is the P value for Bootstrapping method. AIC: Akaike information criterion; NA: Not applicable.

**Supplementary Table S5. Prediction of other liver disease complications using original MELD models.**

|  | **ROC Analysis** | | | | |  |  |  |
| --- | --- | --- | --- | --- | --- | --- | --- | --- |
| **Other complications** | **SEN** | **SPE** | **PPV** | **NPV** | **AUC** | **B(P)** | **HL(P)** | **AIC value** |
| Bacterial peritonitis | NA | NA | NA | NA | NA | NA | NA | NA |
| Encephalopathy | 24.2% | 97.0% | 61.5% | 86.4% | 0.829 | 0.001 | 0.14 | 145.28 |
| GI bleeding | 27.1% | 90.5% | 75.9% | 52.8% | 0.684 | 0.001 | 0.72 | 108.07 |
| Hepatobiliary  carcinoma | NA | NA | NA | NA | NA | NA | NA | NA |
| Hepatorenal. syndrome | NA | NA | NA | NA | NA | NA | NA | NA |
| Portal hypertension | 63.2% | 81.4% | 75.9% | 70.3% | 0.818 | 0.001 | 0.022 | 221.49 |

SEN: Sensitivity; SPE: Specificity; PPV: Positive predictive value; NPV: Negative predictive value; HL: Hosmer–Lemeshow test; AUC: Area under the ROC curve. B(p) is the P value for Bootstrapping method. AIC: Akaike information criterion; NA: Not applicable.
